# Diversity of Dopaminergic Neural Circuits in Response to Drug Exposure

**DOI:** 10.1038/npp.2016.32

**Published:** 2016-04-06

**Authors:** Barbara Juarez, Ming-Hu Han

**Affiliations:** 1 Department of Pharmacology and Systems Therapeutics, Icahn School of Medicine at Mount Sinai, New York, NY, USA; 2 Department of Neuroscience, Icahn School of Medicine at Mount Sinai, New York, NY, USA; 3 Friedman Brain Institute, Icahn School of Medicine at Mount Sinai, New York, NY, USA; 4 Institute for Systems Biomedicine; Icahn School of Medicine at Mount Sinai, New York, NY, USA

## Abstract

Addictive substances are known to increase dopaminergic signaling in the mesocorticolimbic system. The origin of this dopamine (DA) signaling originates in the ventral tegmental area (VTA), which sends afferents to various targets, including the nucleus accumbens, the medial prefrontal cortex, and the basolateral amygdala. VTA DA neurons mediate stimuli saliency and goal-directed behaviors. These neurons undergo robust drug-induced intrinsic and extrinsic synaptic mechanisms following acute and chronic drug exposure, which are part of brain-wide adaptations that ultimately lead to the transition into a drug-dependent state. Interestingly, recent investigations of the differential subpopulations of VTA DA neurons have revealed projection-specific functional roles in mediating reward, aversion, and stress. It is now critical to view drug-induced neuroadaptations from a circuit-level perspective to gain insight into how differential dopaminergic adaptations and signaling to targets of the mesocorticolimbic system mediates drug reward. This review hopes to describe the projection-specific intrinsic characteristics of these subpopulations, the differential afferent inputs onto these VTA DA neuron subpopulations, and consolidate findings of drug-induced plasticity of VTA DA neurons and highlight the importance of future projection-based studies of this system.

## INTRODUCTION

Dopamine (DA) in the mesocorticolimbic system is thought to tune attributes of rewarding stimuli by encoding the value of a reward, creating incentive salience for reward, enhancing associative learning of the reward context, and determining the predictability of a reward (Gonzales *et al*, 2004; Koob, 2006; Robinson and Berridge, 2000; Schultz, 2002). DA in this system has also been shown to be involved in emotion-related behaviors, particularly in the processing of stressful events (Brischoux *et al*, 2009; Chaudhury *et al*, 2012; Tye *et al*, 2013). Ventral tegmental area (VTA) DA neurons of the mesocorticolimbic system release DA onto neural substrates classically known to encode stimuli valence (nucleus accumbens, NAc), regulate executive control (medial prefrontal cortex, mPFC), and form associative related memories (basolateral amygdala, BLA) (Russo and Nestler, 2013). These dopaminergic projections of the VTA are known to be a part of the reward and emotion-related behaviors, which are part of a key neural circuit of addiction (Hyman, 2007; Nestler, 2001). DA neurons of the VTA have also been shown to project to other brain regions, including the hippocampus (HPC), with a particularly heavy distribution in the ventral subiculum (vSub) and CA1 (Gasbarri *et al*, 1994; Scatton *et al*, 1980). This projection has been proposed to be important for long-term potentiation (LTP) and the representation of stimuli and environments (Lisman and Grace, 2005).

**Figure 1:**
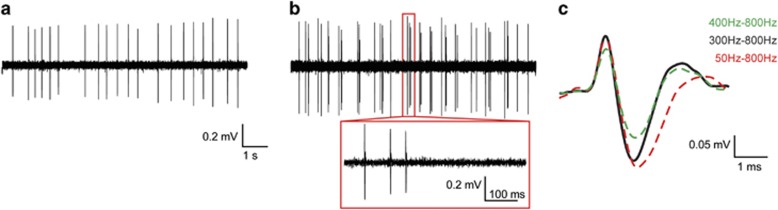
*In vivo* firing characteristics of VTA DA neurons. Active VTA DA neurons transition between two states of firing modes *in vivo*: (a) low-frequency tonic, single-spike firing and (b) high-frequency burst firing (inset shows expanded view of burst with onset occurring when two spikes fire within <80 ms and termination ending after >160 ms of silence). VTA DA neurons have a waveform shape under filter conditions. (c) Demonstration of a single recorded VTA DA neuron under three different filters used in electrophysiology. DA, dopamine; VTA, ventral tegmental area.

VTA DA neurons exhibit two firing patterns *in vivo*—a slow, asynchronous single-spike activity (tonic, 2–4 Hz) and a rapid, multi-spike bursting activity (phasic, 15–30 Hz) (Figure 1) (Cao *et al*, 2010; Grace and Bunney, 1984a, 1984b; Tsai *et al*, 2009). It is thought that transitions between these two modes encode the context of rewarding stimuli, and ultimately the changes in concentration of DA at the terminal help encode salience to stimuli, promote seeking of reward, and tune reward prediction error for cue-associated behaviors (Phillips *et al*, 2003; Schultz, 2007). VTA DA neurons are immunohistochemically identified by the presence of tyrosine hydroxylase (TH), a rate-limiting enzyme in the production of DA (Berger *et al*, 1982; Javoy-Agid *et al*, 1981a; Javoy-Agid *et al*, 1981b; Margolis *et al*, 2006b). The VTA is also comprised of local inhibitory *γ*-aminobutyric acid (GABA) interneurons, projecting GABA neurons and projecting glutamate neurons (Carr and Sesack, 2000a; Lammel *et al*, 2014; Walsh and Han, 2014). In addition, it has been revealed that a subpopulation of midbrain DA neurons projecting to the striatum co-release glutamate and GABA onto their target neural substrates (Kim *et al*, 2015; Stuber *et al*, 2010; Tritsch *et al*, 2012; Tritsch *et al*, 2014). Consequently, understanding the ever-emerging complexity of the VTA and the functions of its projections is a field of great interest.

The VTA is also critical for drug reward, seeking, and reinstatement (Koob and Volkow, 2010). Drugs of abuse increase DA concentrations in projection targets of the VTA and within the VTA itself (Di Chiara and Imperato, 1988; Nestler, 2005; Wise and Rompre, 1989). However, the mechanisms of how drugs of abuse cause increased DA release and increased concentrations of DA in target neural substrates are varied. Drugs of abuse can act directly on VTA DA neurons through receptor binding or ion-channel modulation to increase cell firing, through indirect mechanisms of disinhibition, or through modulation of DA transporters (DAT), which is the main clearance mechanism of DA at the synapse (Hyman *et al*, 2006). Ethanol can act directly on receptors and ion channels located on VTA DA neurons to increase cellular firing while simultaneously modulating GABAergic inputs onto VTA DA neurons (Brodie *et al*, 1990; Gessa *et al*, 1985; Tan *et al*, 2010; Theile *et al*, 2010). Nicotine also directly modulates the activity of VTA DA neurons through binding to a multitude of nicotinic acetylcholine receptors (nAChRs) expressed on DA neurons and glutamatergic terminals in the VTA (Laviolette and van der Kooy, 2004). The overall actions of opioids in the VTA, however, act primarily through inhibitory *μ*-opioid receptors (MORs) expressed on GABAergic interneurons of the VTA, to reduce GABA tone and disinhibit DA neuron firing (Johnson and North, 1992a). The psychostimulant cocaine increases DA concentrations by inhibiting DATs that are present on axon terminals of VTA DA neurons, as well as inducing robust synaptic plasticity mechanisms on VTA DA neurons (Luscher, 2013; Vaughan and Foster, 2013). These drugs demonstrate the multiple mechanisms and complexity by which drugs of abuse modulate DA levels in the mesocorticolimbic system.

Recently, further elucidation of the characteristics and function of the mesocorticolimbic DA circuit has described heterogeneity of VTA DA neurons based on projection-specific, intrinsic receptor/ion-channel distributions and selective afferent inputs (Chaudhury *et al*, 2012; Ekstrand *et al*, 2014; Friedman *et al*, 2014; Lammel *et al*, 2008). These subpopulations can often be anatomically differentiated on a medial-lateral, posterior-anterior, and dorsal-ventral manner (Ford *et al*, 2006; Lammel *et al*, 2008; Margolis *et al*, 2006a; Margolis *et al*, 2006b; Margolis *et al*, 2008). The projection-specific molecular, functional, and anatomical differences must be taken into account when determining the actions of drugs of abuse on DA function. To fully understand the mechanisms of drug-induced plasticity, one must now understand how these subpopulations are uniquely modulated after drug exposure to encode reward salience and cue-induced behaviors that effect drug reinstatement (relapse). This review will describe the intrinsic characteristics of subpopulations of VTA DA neurons, define the differential synaptic control of these subpopulations, and delve into the multivaried mechanisms of drug-induced neuroadaptations that alcohol, nicotine, opiates, and cocaine induce on VTA DA neurons while highlighting studies that have identified atypical VTA DA plasticity mechanisms.

## FUNCTIONALLY DIVERSE INTRINSIC CHARACTERISTICS OF VTA DOPAMINE NEURONS

### Conventional Methods of Identifying VTA Dopamine Neurons *In Vitro*

VTA DA neurons of the mesocorticolimbic system project to the NAc, the mPFC, and the BLA. Since VTA DA neurons lose their characteristic phasic activity in *ex vivo* slice preparations, a number of conventional electrophysiological and functional criteria have been used to identify DA neurons *in vitro* (Grace and Bunney, 1984a, 1984b; Grace and Onn, 1989; Kitai *et al*, 1999). Historically, researchers have used the conventional electrophysiological criteria that were associated with DA neurons of the substantia nigra pars compacta (SNc) to identify DA neurons of the VTA. These criteria included the presence of slow pacemaker firing, a long action potential waveform, a hyperpolarization-activated current (*I*_h_), the presence of small conductance calcium-activated potassium (SK) channels, and autoinhibition through the high-affinity DA D2 receptors that are linked to G protein-coupled inwardly rectifying potassium channels (GIRK) (Liss and Roeper, 2008; Margolis *et al*, 2006b; Morikawa and Morrisett, 2010).

However, DA neurons have proved difficult to conventionally classify in the VTA. *I*_h_ is mediated via hyperpolarization-activated cyclic nucleotide-gated cation channels (HCN channels) and were once thought to be a reliable criterion for functional identification of DA neurons. However, although DA neurons of the SNc consistently display *I*_h_ to maintain pacemaker activity, VTA DA neurons have shown great heterogeneity in the expression and function of *I*_h_ (Liss and Roeper, 2008; Margolis *et al*, 2010; Margolis *et al*, 2006b). These investigations have varied in the rodent models used (rat or mouse), age of model (adolescent or adult), and recording conditions (room temperature or 32 °C), yet they have consistently identified a posterior-medial subpopulation of putative TH-expressing DA neurons in the VTA that display little or no *I*_h_ currents in acute slice preparations (Ford *et al*, 2006; Friedman *et al*, 2014; Lammel *et al*, 2008; Margolis *et al*, 2006b). This posterior-medial subpopulation of VTA DA neurons have been consistently shown to project to the mPFC and also display higher baseline activity profiles (Lammel *et al*, 2008). Another marker of DA neurons in the SNc was the presence of apamin-sensitive SK channels (Liss and Roeper, 2008). These channels have been shown to generate the distinguishing after hyperpolarization of VTA DA neuron waveforms (Ji *et al*, 2009; Ji and Shepard, 2006). It is also a channel critical for the phasic activity pattern generation of DA neurons in the SNc (Ji and Shepard, 2006; Liss and Roeper, 2008). However, SK channels were also inconsistently found across the DA neurons of the VTA. Interestingly, low SK-mediated currents were correlated with little to no *I*_h_ currents (Table 1) (Lammel *et al*, 2008). Furthermore, there have been a number of reports of discrepancies in using D2 receptor-mediated autoinhibition to identify VTA DA neurons. This autoinhibition mechanism is regulated by the presence of somatodendritic D2 receptors that are linked to GIRK channels on DA neurons. Activation of D2 receptors, either through localized DA release in the VTA or the D2 receptor agonist, quinpirole, leads to hyperpolarization of the cell to inhibit firing (Beckstead *et al*, 2004; Labouebe *et al*, 2007). However, subpopulations of VTA DA neurons showed no inhibition effect to activation of these receptors (Chiodo *et al*, 1984). The disparate presence of these classic criteria in VTA DA neurons has called into question what constitutes the classic characteristics of DA neurons in the VTA.

### Intrinsic Heterogeneity Based on Projection Site

**Figure 2:**
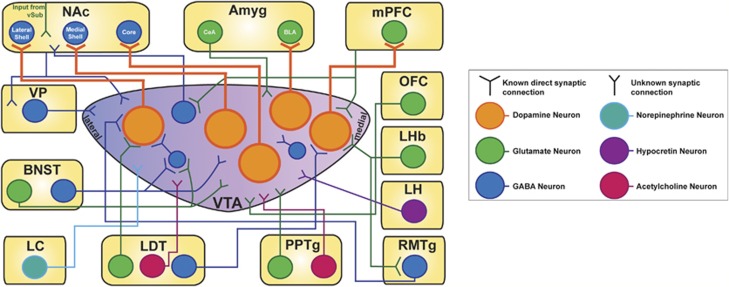
Schematic representation of VTA input and output. Amyg, amygdala; BNST, bed nucleus of stria terminalis; LH, lateral hypothalamus; LC, locus coeruleus; LDT, laterodorsal tegmental nucleus; LHb, lateral habenula; mPFC, medial prefrontal cortex; NAc, nucleus accumbens; OFC, orbitofrontal cortex; PPTg, pedunculopontine tegmental nucleus (aka PPN); RMTg, rostral medial tegmental nucleus; VP, ventral pallidum; vSub, ventral subiculum; VTA, ventral tegmental area.

**Figure 3:**
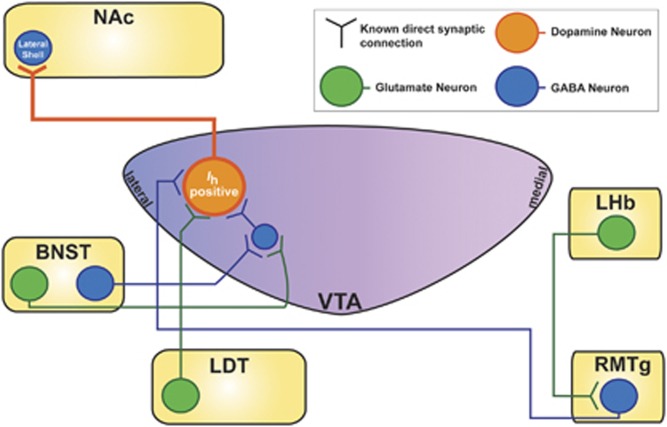
Known inputs onto *I*_h_-positive VTA DA neurons. VTA DA neurons that project to specific regions of the mesocorticolimbic system can often be identified by the presence and absence of an *I*_h_ current. This figure shows *I*_h_-positive circuits. BNST, bed nucleus of stria terminalis; DA, dopamine; *I*_h_, hyperpolarization-activated cation channel current; LDT, laterodorsal tegmental nucleus; LHb, lateral habenula; NAc, nucleus accumbens; RMTg, rostral medial tegmental nucleus; VTA, ventral tegmental area.

**Figure 4:**
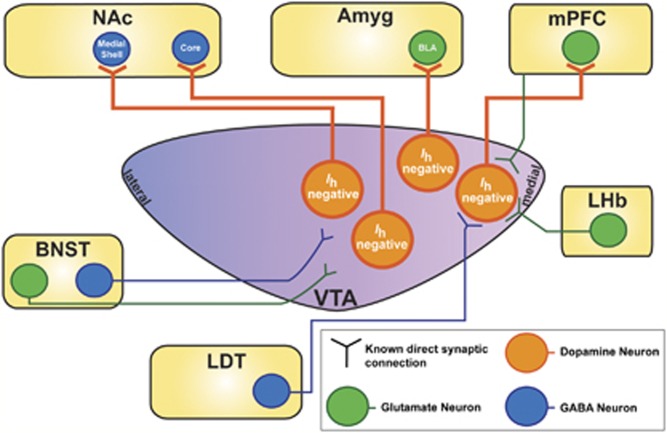
Known inputs onto *I*_h_-negative VTA DA neurons. This figure shows known direct synaptic inputs onto *I*_h_-negative neurons and their projection targets. Amyg, amygdala; BNST, bed nucleus of stria terminalis; DA, dopamine; *I*_h_, hyperpolarization-activated cation channel current; LDT, laterodorsal tegmental nucleus; LHb, lateral habenula; mPFC, medial prefrontal cortex; NAc, nucleus accumbens; RMTg, rostral medial tegmental nucleus; VTA, ventral tegmental area.

Using circuit-tracing techniques, there have been a number of recent studies that have elucidated distinct subpopulations of VTA DA neurons of the mesocorticolimbic system based on their projection site and TH immunoreactivity. VTA DA neurons with large *I*_h_ were mostly located in the lateral VTA and project to the lateral shell of the NAc (lsNAc) (Table 1, Table 2, Figure 2, and Figure 3) while VTA DA neurons that had little to no *I*_h_ were found to be primarily distributed along the posterior-medial axes of the VTA (Table 1, Figure 2, and Figure 4). VTA DA neurons with little or no *I*_h_ project to the mPFC, the BLA, the NAc core (cNAc), and medial shell of the NAc (msNAc) (Table 1 and Figures 2 and 4). They also displayed higher firing rates than the typical slow pacemaker activity patterns previously attributed to VTA DA neurons, had increased membrane excitability, and showed differential homeostatic plasticity mechanisms (Table 1) (Chaudhury *et al*, 2012; Friedman *et al*, 2014; Lammel *et al*, 2008). Uniquely, VTA DA neurons that project to the mPFC (VTA-mPFC) lacked D2 receptor-mediated autoinhibition and display lower levels of D2 and GIRK channel mRNA (Table 1) (Lammel *et al*, 2008). These VTA-mPFC neurons also have a very low expression of DAT when compared with mesolimbic projecting neurons (Table 1) (Lewis *et al*, 2001; Sesack *et al*, 1998). Indeed, it has been shown that the mPFC maintains higher concentrations of DA for longer amounts of time compared with the striatum (Garris *et al*, 1993; Moghaddam *et al*, 1993). This decreased uptake for DA in VTA-mPFC DA neurons could be of functional importance for the DA in working memory and executive behaviors in the cortex.

In conjunction with reduced uptake and D2 autoreceptors on VTA dopaminergic terminals in the mPFC is a differential DA metabolic turnover mechanism compared with subcortical regions (Bannon and Roth, 1983). The enzyme catechol-*o*-methyltransferase (COMT) has a role in the metabolism of DA in the PFC (Bilder *et al*, 2004; Guldberg and Marsden, 1975). COMT downregulation leads to increased DA in the PFC, but not the NAc (Gogos *et al*, 1998; Huotari *et al*, 2002). COMT-knockout (KO) mice have increased DA levels in the PFC *in vivo*, but not in subcortical regions, when blocking norepinephrine transporters (NET) and monoamine oxidation (MAO), suggesting a critical role of COMT and its regulation with NET and MAO in differential DA clearance rates in the PFC (Kaenmaki *et al*, 2010).

If VTA DA neurons in early drug-induced plasticity studies were once classified based on the conventional criteria of the presence of a large *I*_h_ or D2 receptor-mediated autoinhibition, those studies might have preferentially identified plasticity mechanisms of VTA DA neurons that projected exclusively to the specific subcortical regions. This should be taken into account when interpreting earlier work of studying drug-induced plasticity that based their identification of DA neurons *in vitro* on these criteria. Indeed, functional encoding of depressive behaviors were found to be different amongst subpopulations of VTA DA neurons (Chaudhury *et al*, 2012) and a homeostatic plasticity mechanism of balancing potentiated *I*_h_ and inhibitory potassium (K^+^) currents, associated with resilience to stress, were found to be exclusively occurring in VTA-NAc DA neurons (Friedman *et al*, 2014). Following this discovery of projection-specific stress-related adaptations, it is possible that drugs of abuse, which act via ion-channel modulation, receptor binding, or DAT inhibition, may also induce heterogeneous adaptations across projection-specific subpopulations. Thus, understanding possible differential mechanisms of drug-induced plasticity across distinct VTA DA populations could support the multivaried roles of DA found in the mesocorticolimbic system. Below, we will discuss our current understanding of drug-induced plasticity mechanisms and try to dissociate the findings based on each subpopulation.

## A CIRCUIT-LEVEL VIEW OF NEURAL FUNCTION AND BEHAVIORAL CONTROL OF VTA DOPAMINE NEURONS

### *In vivo* Characteristics of VTA Dopamine Neurons

VTA DA neurons can be reliably identified *in vivo* by their slow waveform of >2.5 ms (Figure 1a and c) and large start-negative trough segment of ⩾1.1 ms (Figure 1c) (Grace and Bunney, 1983a, 1983b; Henry *et al*, 1989; Ungless and Grace, 2012; Ungless *et al*, 2004). VTA DA neurons are known to express and transition between two firing states *in vivo*, a single-spike tonic firing (1–10 Hz) and a transient, multi-spike phasic firing (15–30 Hz) (Figure 1b) (Cao *et al*, 2010; Grace and Bunney, 1984a, 1984b; Grace and Onn, 1989; Walsh and Han, 2014). These two active states contribute to the tonic-phasic DA hypothesis where tonic VTA DA activity sets a low background DA level in downstream regions, whereupon the concentration of DA is significantly elevated with behaviorally significant stimuli (Bilder *et al*, 2004; Floresco *et al*, 2003).

Modifying filter parameters during recording can alter the waveform of putative VTA DA neurons (Figure 1c). Although sampling a large number of neurons can eliminate the possibility of misidentification of VTA DA neurons *in vivo*, the risk of misidentification in smaller neuronal samples could be ameliorated with stringent filtration settings during recording, followed by immunohistochemical verification for the presence of TH after recording (Ungless and Grace, 2012; Ungless *et al*, 2004). In addition, monitoring D2 receptor-mediated autoinhibition is also a way to identify VTA DA neurons *in vivo*, but excludes a subpopulation of VTA DA neurons that do not have strong autoinhibition (Chiodo *et al*, 1984; Lammel *et al*, 2008).

### Glutamate and GABA as Regulators of Dopaminergic Firing

Regulation of tonic and phasic firing *in vivo* is known to be mediated by the coordination of a number of glutamatergic, GABAergic and cholinergic afferent inputs onto VTA DA neurons (Figure 2). Activation of ionotropic glutamate receptors, including *N*-methyl-D-aspartate receptor (NMDA) receptors and *α*-amino-3-hydroxy-5-methyl-4-isoxazolepropionic acid (AMPA) receptors on VTA DA neurons increases VTA DA neuron *in vivo* firing rate and bursting activity (Chergui *et al*, 1993; Georges and Aston-Jones, 2002; Johnson *et al*, 1992b; Zweifel *et al*, 2009). The VTA receives glutamate control from various cortical substrates. For example, VTA dopaminergic and non-dopaminergic neurons receive input from the orbitofrontal cortex (OFC) (Figure 2), a key region for the encoding of reward value and prediction error (Roesch *et al*, 2007; Takahashi *et al*, 2011). Lesioning the OFC does not affect VTA DA firing, but does cause reduced firing in non-dopaminergic VTA neurons (Takahashi *et al*, 2011). OFC-lesioned rats showed reduced phasic responses to unexpected reward, no suppression of firing to reward-omitted events, and weaker responses to cues that predicted the value of the reward (Takahashi *et al*, 2011). However, stimulating the OFC caused inhibition in 73% of recorded VTA DA neurons, no change in 13% of recorded VTA DA neurons, and excitation in 13% of recorded VTA DA neurons, demonstrating heterogeneity in OFC synaptic modulation and supporting the idea of a multisynaptic circuit of control (Takahashi *et al*, 2011).

The PFC has also been shown to send glutamatergic afferents to the VTA to control burst firing (Figure 2) (Gariano and Groves, 1988; Sesack and Bunney, 1989; Tong *et al*, 1996b). Later studies have demonstrated the importance of PFC glutamatergic input for the modulation of burst activity in VTA DA neurons. Electrical stimulation of the PFC was shown to induce bursting in the VTA, which is inhibited with ionophoretic application of the NMDA antagonist CPP, but not the AMPA antagonist CNQX, onto VTA DA neurons (Tong *et al*, 1996a). Interestingly, Gariano and Groves, 1988 reported an initial brief inhibition followed by activation of VTA burst activity with PFC stimulation. Tong *et al* hypothesized that Gariano and Groves observed an additional cellular mechanism of PFC-induced bursting after the discovery of heterogeneous burst responses in the VTA following PFC electrical stimulation. Using physiologically relevant electrical stimulation parameters, they identified one class of neurons that would burst due to an initial excitation (named ‘E', 27.9% of VTA DA neurons recorded) and another class of neurons that would burst due to excitation following a short inhibition (named rebound ‘IE', 33.3% of VTA DA neurons recorded). The mechanism of bursting in the IE subpopulation was hypothesized to be due to rebound activation of a low threshold calcium current on these neurons, similar to those neurons in the thalamus (Tong *et al*, 1996b). The mechanism of bursting on the E population was found to be directly produced by glutamate. Importantly, they noted that VTA DA neurons had the ability to switch their characteristics from E to IE or from IE to E, depending on the current used, highlighting the role of and significance of PFC-VTA regulation (Sesack and Carr, 2002; Tong *et al*, 1996b). These differential responses to PFC stimulation and OFC ablation further highlight the heterogeneity of VTA DA neuron subpopulation connectivity.

In addition to glutamatergic inputs from cortical regions, the VTA also receives a number of excitatory inputs from subcortical brain structures that may be relevant to the integration of environmental stimuli. The extended amygdala, including the bed nucleus of stria terminalis (BNST) and the central amygdala (CeA), both send glutamatergic projections to the VTA (Figure 2) (Geisler and Zahm, 2005; Georges and Aston-Jones, 2001; Gonzales and Chesselet, 1990; Vanderschuren and Kalivas, 2000; Wallace *et al*, 1992). It has been demonstrated that the BNST can act as mediator between the vSub and the VTA to control firing (Jalabert *et al*, 2009). In addition, cholinergic and glutamatergic signaling in the VTA has been shown to be critical to regulating burst activity of DA neurons. The pedunculopontine tegmental nucleus (PPTg, also known as PPN) sends glutamatergic and cholinergic inputs to the VTA (Figure 2) (Clements and Grant, 1990; Oakman *et al*, 1995). Increasing activity from the PPTg was shown to selectively increase bursting activity in already active VTA DA neurons to lead to increased concentrations of DA in the NAc (Floresco *et al*, 2003). In mice lacking NMDA receptors in VTA DA neurons, the PPTg no longer drove burst activity (Zweifel *et al*, 2009). In addition, glutamatergic and cholinergic afferents from the laterodorsal tegmental nucleus (LDT) were found to be critical regulators of VTA DA phasic activity and consequent increased DA release in the NAc (Figure 2) (Faure *et al*, 2014; Forster and Blaha, 2000; Lodge and Grace, 2006).

In addition to inhibitory control from VTA GABAergic interneurons, VTA DA neurons also receive inhibitory afferents from a number of regions that can control VTA DA activity *in vivo* (Figure 2). Using retrograde tracing techniques, investigators discovered that there are strong GABAergic innervations originating from the NAc shell and the ventral pallidum (VP) (Figure 2) (Kalivas *et al*, 1993; Wu *et al*, 1996; Zahm and Heimer, 1990). Eliminating the GABAergic tone from the VP by activating the hippocampal vSub-NAc-VP loop was found to increase the number of asynchronous tonic firing DA neurons in the VTA, without affecting overall increases in firing rate or bursting activity, suggesting that GABAergic VP afferents control a number of quiescent cells (Figure 2) (Floresco *et al*, 2003). A region found caudal to the VTA, the rostral tegmental nucleus (RMTg), has also been observed to be a major inhibitory source for dopaminergic neurons in the SNc and the VTA (Figure 2) (Barrot, 2014; Sanchez-Catalan *et al*, 2014).

### Differential Afferent Synaptic Control Over Subpopulations of VTA DA Neurons

Importantly, studies have demonstrated that not all VTA DA neurons receive the same afferent inputs, and often these VTA DA neurons can be dissociated from one another based on the presence of *I*_h_, their projection site or regional distributions across the VTA (Figures 3 and 4, and Table 2). The glutamatergic afferents from the PFC synapse differently on cellular populations in the VTA. DA neurons that project to the mPFC, a typically *I*_h_-negative population, receive direct excitatory glutamatergic feedback from the PFC (Figure 4) (Carr and Sesack, 2000b). VTA GABAergic projecting neurons to the NAc also receive glutamatergic control from the PFC (Carr and Sesack, 2000b).

There are also dissociable afferent synapses from subcortical regions onto VTA DA subpopulations. Studies showed that LDT excitatory afferents synapse specifically on VTA DA neurons projecting to the NAc (VTA-NAc) (Figure 3) (Lammel *et al*, 2011; Omelchenko and Sesack, 2005). Conversely, VTA DA neurons projecting to the mPFC (VTA-mPFC) receive inhibitory GABAergic afferents from the LDT (Figure 3) (Omelchenko and Sesack, 2005). Additionally, norepinephrine (NE) inputs from the locus coeruleus (LC) were demonstrated to be distributed across the VTA, with denser varicosities distributed across the medial (central) portions of the VTA (Figure 2) (Mejias-Aponte *et al*, 2009). Recently, an extensive tracing study revealed input-output connections of VTA DA neurons (Beier *et al*, 2015).

Understanding how cortical and subcortical afferents onto subpopulations of VTA DA neurons are differentially altered after drug exposure from a circuit perspective could generate a new understanding of drug-motivated behaviors. The synaptic strength of glutamatergic signals onto VTA DA neurons can be observed *in vitro* by measuring the currents mediated by ionotropic AMPA and NMDA glutamate receptors. AMPA receptors are tetrameric proteins that can be composed of four different types of subunits. AMPA receptors are quickly activated by glutamate and help depolarize the cell with the passage of small cations. This depolarization in turn helps activate the voltage-sensitive glutamate NMDA receptors, which passes small cations and, importantly, calcium. This increase in calcium influx is important for activation of many second messenger systems and for the formation of longer lasting plasticity. The functions between these two receptors can be measured *in vitro* by measuring currents mediated by AMPA and NMDA receptors and creating an AMPA/NMDA ratio. Alteration of this AMPA/NMDA ratio is one measure of drug-induced synaptic plasticity, as it alludes to the effectiveness of glutamatergic plasticity mechanisms on AMPA receptors and NMDA receptors.

In addition, another measure of synaptic strength is the rate of induction of LTP, using high-frequency stimulations, or long-term depression (LTD), using low-frequency stimulation, at glutamatergic or GABAergic synapses. Since the subpopulations of VTA DA neurons receive unique glutamatergic and GABAergic afferent modulation, it is critical to first identify the source of glutamatergic or GABAergic signals on a subpopulation of VTA DA neurons using advanced circuit-tracing techniques, then observing and altering the strength of these signals after drug exposure using optogenetics to see how a circuit is altered. The differential afferent input control highlights the possibility that subpopulations of VTA DA neurons can serve specific functions in behavioral regulation, particularly because of the diverse roles these regions have in processing environmental stimuli. There is now a critical need to understand these circuits, particularly to identify specific mechanisms of drug-induced plasticity.

### Neuromodulators and Their Actions on VTA DA Neurons

DA neurons receive modulatory inputs from substrates that release a number of neuropeptides and steroid hormones. These modulators have slow and long-lasting actions through G protein-coupled receptors, to activate second messenger systems in neurons. VTA DA neurons express a number of these receptors, which ultimately influence synaptic communication between fast-acting excitatory and inhibitory afferents. Here, we will discuss the varied roles that the neuromodulators hypocretins (hcrt, also known as orexins), corticotrophin releasing factor (CRF) and glucocorticoids have in modulating VTA DA neural activity.

In addition to sending GABAergic and glutamatergic projections to the VTA, the lateral hypothalamus (LH) sends robust hcrt input to the VTA (Figure 2) (Fadel and Deutch, 2002; Peyron *et al*, 1998). The LH synthesizes hypocretin-1 (hcrt-1/orexin A) and hypocretin-2 (hcrt-2/orexin B) (Peyron *et al*, 1998; Sakurai *et al*, 1998). Hcrt-1 has a higher affinity for Gq-coupled receptor hypocretin receptor-1 (hcrt-R1); hcrt-2 shows equal affinity for hcrt-R1 and hcrt-2R, which is coupled to Gq and Gi/o receptors (Sakurai *et al*, 1998; Zhu *et al*, 2003). The VTA is known to express both receptors (Korotkova *et al*, 2003; Narita *et al*, 2006). *In vivo* infusion of hcrt-1 into the VTA was shown to increase DA release in the mPFC and mNAc, but not the NAc core (Narita *et al*, 2006; Vittoz and Berridge, 2006). It has been suggested that this activation of DA release could be mediated through hcrt-1's ability to potentiate NMDAR transmission (Borgland *et al*, 2006; Morikawa and Morrisett, 2010). Modulating the hcrt system in awake and behaving animals has been known to affect arousal (hcrt-2) and strengthen cued-reinstatement behaviors (Korotkova *et al*, 2006; Smith *et al*, 2009). Importantly, hcrts have been shown to have a critical role in mediating the associative and cue-induced reinstatement properties of multiple classes of drugs, including alcohol, nicotine, morphine, and cocaine (Harris *et al*, 2005; Hollander *et al*, 2008; Lawrence *et al*, 2006; Narita *et al*, 2006). The observed role of hcrts in mediating DA release, VTA DA synaptic activity and reward seeking makes it an important neuropeptide in the study of drug-induced plasticity.

Interestingly, there have been numerous reports of heterogeneous responses to hcrt in the VTA. Hcrt-1 infusion *in vivo* preferentially activated DA neurons of the caudomedial VTA, as determined by c-fos immunoreactivity (Vittoz *et al*, 2008). Korotkova *et al*, 2003 identified three classes of DA neurons after application of hcrt-1 in acute VTA brain slices: one showed tonic activation; another showed inactivation; and a third showed bursting-like activation in slice. These differential, and possibly subpopulation-specific effects, of hcrt signaling via the LH could be informative to the dissection of subpopulations on VTA DA neurons.

The VTA is also an important target for the stress-related neuropeptide, CRF (sometimes referred to as corticotrophin releasing hormone-CRH) (Swanson *et al*, 1983). The VTA receives CRF from projecting terminals of the BNST, the CeA, the paraventricular nucleus of the hypothalamus (PVN), and even from local CRF synthesizing neurons in the VTA (Grieder *et al*, 2014; Korotkova *et al*, 2006; Rodaros *et al*, 2007). This diversity of CRF input into the VTA highlights the VTA's role of integrating environmental and intrinsic cues for behavioral output. CRF binds to two types of G protein-coupled receptors, CRF-R1 (high affinity) and CRF-R2 (low affinity), both of which are expressed in the VTA (Sauvage and Steckler, 2001; Ungless *et al*, 2003; Van Pett *et al*, 2000). In addition, CRF-binding protein (CRF-BP) was found to be critical for CRF's actions in the VTA (Ungless *et al*, 2003). CRF containing terminals make both asymmetric (excitatory) and symmetric (inhibitory) synapses in the VTA on both dopaminergic and non-dopaminergic cells (Tagliaferro and Morales, 2008). Interestingly, in the same anatomical investigation, the experimenters discovered that only a subpopulation of VTA DA neurons expressed CRF-BP, highlighting again the importance of elucidating the heterogeneous mechanisms of action of VTA DA neurons. CRF's role in stress-related, drug-induced plasticity and behaviors will be discussed further below.

The neuropeptide CRF/CRH from the hypothalamus also causes the activation of the stress-reactive hypothalamus-pituitary-adrenal (HPA) pathway, which ultimately releases corticosteroids from adrenal glands. Dysregulation of this HPA activation can lead to a host of neuropsychiatric disorders (de Kloet *et al*, 2005). One type of corticosteroid, glucocorticoids (GCCs), has potent actions in the mesocorticolimbic system during stressful events (Piazza and Le Moal, 1996). Low concentrations of GCCs bind with high affinity to mineralocorticoid receptors (MRs) while the lower affinity glucocorticoids receptors (GRs) are activated during times of higher GCC release (McEwen *et al*, 2015). Adrenalectomy reduces DA release in the lsNAc and injection of corticosterone to mimic GCC replacement restored DA function (Barrot *et al*, 2000). Differential effects of GCC modulation on DA release are dependent on the severity of stress (acute *vs* chronic) (Marinelli and Piazza, 2002; Pacak, 2000). GCCs also act to regulate VTA DA neural activity to through MR regulation of glutamate release and subsequent NMDA-dependent burst firing (Overton *et al*, 1996). Functioning GRs were determined to be necessary for the stress-induced enhancement of AMPA/NMDA ratio on VTA DA neurons (Saal *et al*, 2003). Moreover, socially defeated mice with intact GR expression in the VTA exhibit increased firing frequency and bursting, while VTA DA neurons in mice without GRs in D1 neurons fail to mount a hyperexcitable response, suggesting a GR-dependent interaction between dopaminoceptive and dopaminergic populations in the mesocorticolimbic circuit following stress (Barik *et al*, 2013). The biphasic modulation of GCCs, the necessity of glutamatergic signaling and the control of VTA DA firing between GCC responsive neural substrates of the emotion-related circuit demonstrate the tightly regulated mechanisms by which GCCs modulate DA dynamics in the mesocorticolimbic system.

### The VTA Processes Both Appetitive and Aversive Events

Increases in VTA dopaminergic signaling is known to be critical for signaling the attributes of rewarding or appetitive stimuli (Hamid *et al*, 2015; Schultz, 2002). Interestingly, VTA DA neurons also serve as a substrate for signaling stressful or aversive events through changes in phasic activity (Brischoux *et al*, 2009; Chaudhury *et al*, 2012; Matsumoto and Hikosaka, 2007). Classically, VTA DA neurons were thought to be inhibited during aversive stimuli (Schultz *et al*, 1997). However, evidence has suggested that aversive stimuli have diverse effects on the regulation of VTA DA subpopulation neuronal activity. Both chronic restraint stress in rats and chronic social defeat stress in mice have been shown to induce long-term increases in VTA DA activity and bursting *in vivo* (Anstrom and Woodward, 2005; Chaudhury *et al*, 2012; Valenti *et al*, 2011). Conversely, rapidly inhibiting VTA DA neuron activity was demonstrated to relieve depressive symptoms (Chaudhury *et al*, 2012). The increased *in vivo* activity observed in VTA DA neurons following social defeat was found in an *in vitro* preparation to be exclusive to the population of DA neurons projecting to the NAc; VTA neurons projecting to the mPFC displayed hypoactivity following social defeat stress (Chaudhury *et al*, 2012). Interestingly, footshock stress has been shown to rapidly inhibit or activate subpopulations of VTA DA neurons (Brischoux *et al*, 2009; Valenti *et al*, 2011). A population of those inhibited neurons were observed to show increased firing at the termination of the footshock, suggesting the termination of aversive stimuli was found to be rewarding (Brischoux *et al*, 2009). On the molecular level, NMDA receptors were found to be critical to the activation of VTA DA neurons in aversive situations (Zweifel *et al*, 2011). These studies highlight the discovery of VTA DA subpopulations in the response to stress and aversive stimuli.

The differential glutamatergic and GABAergic synaptic connections described above may underlie the ability of the VTA to respond to both appetitive and aversive stimuli. Interfering with ventral HPC (vHPC)/vSub circuits have demonstrated that the vHPC region is critical in modulating the population of active and inactive VTA DA neurons. Inactivating the vHPC prevents or reverses the observed footshock and restraint stress increases in population activity found in the subset of VTA DA neurons (Valenti *et al*, 2011).

With the advent of circuit dissecting techniques, a number of studies have delved into dissociating the appetitive and aversive behavioral responses mediated by GABAergic and glutamatergic afferents onto VTA DA neurons. The BNST sends divergent glutamatergic and GABAergic projections to the *I*_h_-positive or -negative VTA neurons to encode aversive properties uniquely based on their medial to lateral synaptic targets (Figures 3 and 4) (Jennings *et al*, 2013). In addition to the BNST, Lammel *et al* demonstrated that LDT glutamatergic afferents synapse onto lateral VTA (lVTA) DA neurons that project to the lsNAc (Figure 3). Optogenetically activating this LDT-lVTA-lsNAc circuit in one chamber during training sessions of conditioned place preference (CPP) caused a strong preference for the optically stimulated side (Lammel *et al*, 2012). In contrast, glutamatergic synapses originating from the lateral habenula (LHb) can synapse on medial VTA (mVTA)-mPFC DA neurons or onto GABAergic RMTg neurons that then project to lVTA-lsNAc neurons or medial PFC (Figures 3 and 4); optically stimulating glutamatergic LHb afferents that project to mVTA neurons or RMTg-lVTA neurons caused significant conditioned place aversion (Lammel *et al*, 2012).

The heterogeneous responses of VTA DA neuron subpopulations to appetitive and aversive stimuli and their differential regulation by cortical and subcortical inputs highlight the critical need for the dissection of functionally distinct VTA DA neuron circuits. The molecular basis of this encoding has been recently discussed in a thorough review (Pignatelli and Bonci, 2015). Below, we will discuss the ability of the VTA to process both stressful and rewarding events to modulate drug-induced plasticity mechanisms.

## ACTIONS AND BEHAVIORAL EFFECTS OF DRUGS OF ABUSE

Drug addiction is characterized as the compulsive use of a drug, even in the face of adverse consequences. This pathological behavior forms after repeated use of a drug and subsequent adaptations in many circuits of the brain. Intense investigations in both animal models and humans throughout the years have revealed a critical role for the dopaminergic signaling in the mesocorticolimbic system in the induction of these behaviors. VTA DA neurons undergo adaptations to acute and chronic use of drugs. Below, we will review the way alcohol, nicotine, opiates, and cocaine employ unique mechanisms to alter DA signaling and cause drug-induced plasticity. In addition, we will review investigations that answer how the neuromodulators act to potentiate drug-induced plasticity of VTA DA neurons.

VTA DA neurons are not a homogeneous population and can undergo distinct, projection-specific adaptations. Most of the methods used to identify VTA DA neurons in the reviewed studies used conventional criteria to identify VTA DA neurons, which the field now knows is not standard for all subpopulations. We will thus highlight studies that have found subpopulations of VTA DA neuron that underwent unique plasticity mechanisms. In addition, most of the studies in this review describe the mechanisms from passively administered drug investigations. However, there exist unique mechanisms between investigator-administered drugs of abuse and volitional consumption or self-administration. We will highlight the investigations that observed plasticity mechanisms during self-administration, particularly during the review of how stress influences drug-induced plasticity and relapse behaviors.

### Intrinsic and Synaptic Actions on the Activity of VTA DA Neurons in Response to Ethanol

**Figure 5:**
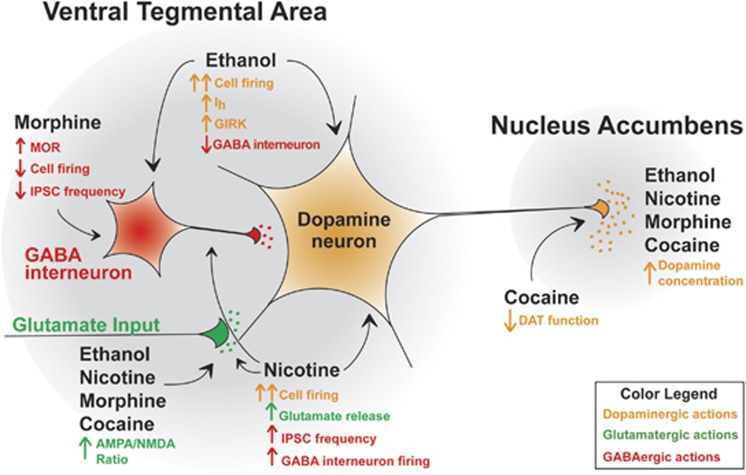
Drugs of abuse act in different ways to increase DA concentrations in downstream targets of the VTA. Pictured here is a VTA DA neuron projecting to the NAc. Ethanol acts directly on VTA DA neurons to increase VTA cell firing, increase *I*_h_, and increase GIRK activity. This increase in activity leads to increased DA release. Ethanol also has effects on VTA GABA interneurons. Nicotine acts on VTA DA neurons themselves through the ligand-gated ion channel, nAChRs. Nicotine also has effects on GABA interneurons and glutamatergic afferents. Morphine acts indirectly through GABA interneurons to increase VTA DA activity. Morphine binds to MORs to decrease IPSC frequency and decrease GABAergic cell firing. This decrease in GABAergic tone disinhibits VTA DA neurons, increasing DA concentrations in the NAc. Cocaine increases concentrations of DA in the NAc by inhibiting DAT function at the VTA DA terminal. Interestingly, acute administration of ethanol, morphine, and cocaine was shown to enhance glutamatergic synaptic strength, as seen through an increase AMPA/NMDA ratio. DA, dopamine; DAT, dopamine transporter; GIRK, G protein-coupled inwardly rectifying potassium channels; *I*_h_, hyperpolarization-activated cation channel current; IPSC, inhibitory post-synaptic current; MOR, *μ*-opioid receptor; NAc, nucleus accumbens; VTA, ventral tegmental area.

Alcohol addiction is marked by a person's transition from casual alcohol consumption to pathological and compulsive alcohol drinking behaviors. This transition is evidenced by a number of plasticity events that regulate reward and behavior. The VTA is a critical neural substrate for the rewarding properties of alcohol (Koob and Volkow, 2010; Nestler, 2005). Administration of ethanol (EtOH) during *in vivo* recordings causes an increase in the activity of VTA DA neurons (Gessa *et al*, 1985). In addition, EtOH administration increases DA levels in VTA target regions of the mesocorticolimbic system (Di Chiara and Imperato, 1985; Weiss *et al*, 1993). The mechanisms of this increased release have been widely studied and have mostly been attributed to EtOH's actions on dopaminergic cell bodies, rather than actions on dopaminergic terminals. Superfusion of EtOH in acute brain slices from rats was shown to increase firing in 89% of the VTA DA neurons recorded in a concentration-dependent manner (Brodie *et al*, 1990). In addition, investigators have identified that in the population of EtOH responsive neurons, there was variability in the sensitivity to the excitation (Brodie *et al*, 1990). Importantly, even after synaptic inputs were blocked, EtOH continued to increase VTA DA activity (Brodie *et al*, 1990; Okamoto *et al*, 2006). Furthermore, VTA DA neurons in isolated cell culture preparations exposed to EtOH still increased their neural activity (Brodie *et al*, 1999). These studies support the idea that EtOH can act directly through intrinsic properties on VTA DA neurons to increase cell firing (Figure 5).

Chronic EtOH exposure can produce compensatory intrinsic plasticity changes in the mesolimbic system to counter the excitatory actions of EtOH and promote EtOH seeking (Weiss and Porrino, 2002). Interestingly, two studies showed disparate responses (sensitization and tolerance) to the excitatory properties of EtOH in acute VTA slice preparations after chronic EtOH exposure. Sensitization to a drug refers to the drug's enhanced effects than previously described after intermittent or chronic exposure. In contrast, developing tolerance to a drug of abuse refers to the drug's inability to induce the same responses in the reward system as it once did; often, higher concentrations or continuous exposure are needed to induce the effects of tolerance. One study described that after chronic EtOH (3.5 mg/kg twice daily i.p. injections for 21 days), VTA DA neurons had become sensitized and displayed increased firing rate and responded more robustly to superfusion of increasing concentrations of EtOH *in vitro* than in saline-treated mice (Brodie, 2002). However, Okamato *et al* showed that 5 days of 2 mg/kg i.p. EtOH injection decreased the EtOH-induced excitability. They attributed this tolerance effect to less effective *I*_h_ (Okamoto *et al*, 2006). These confounding results might be explained by the time course of the injections, 5 *vs* 21 days.

It is known that *in vivo* activity of VTA DA neurons is significantly decreased during withdrawal (Diana *et al*, 1993). In this withdrawal state, there is also a significant reduction of DA release in the NAc (Weiss *et al*, 1996). One leading hypothesis is that this transition into a hypodopaminergic state within the mesocorticolimbic circuit drives the subject to seek more EtOH to stimulate DA release. Indeed, one study found that self-administration of ethanol continued until DA concentrations in the NAc were normalized (Weiss *et al*, 1996). It is critical to fully investigate how plasticity mechanisms over time alter VTA DA neurons, to have a better understanding of how the dopaminergic reward system alters its properties across the different stages of addiction.

EtOH's varied affects are mediated via actions on intrinsic properties through modulation of ion channels and receptors of VTA DA neurons. It has been reported that EtOH directly binds to GIRK channels to increase their function, without the need for G protein-coupled receptor activation (Figure 5) (Aryal *et al*, 2009; Bodhinathan and Slesinger, 2014). In addition, to identify a possible mechanism for the increase in dopaminergic firing in the VTA, Morikawa and colleagues have performed extensive investigations in C57BL/6J mice into how HCN channels that regulate *I*_h_ might control this firing change. They found that EtOH increased levels of *I*_h_ and that blockade of *I*_h_ via bath application ZD7288 significantly reduced the excitatory actions of superfusion of ETOH on VTA DA neurons (Figure 5) (Okamoto *et al*, 2006). In addition to actions on HCN channels, chronic EtOH also has actions on D2-mediated autoinhibition of VTA DA neurons. One day after chronic EtOH administration (2 mg/kg i.p. injection three times a day for 7 days), D2 receptor-mediated autoinhibition was greater and these neurons showed less desensitization (Perra *et al*, 2011). This, in turn, caused a greater decrease in firing after D2 activation with quinpirole (Perra *et al*, 2011), suggesting a functional alteration of VTA DA neurons following chronic EtOH exposure. EtOH's excitatory effects on VTA DA neurons was also shown to be modulated through regulation of SK channels (Brodie *et al*, 2007). Repeated administration of EtOH (2 g/kg i.p injections twice daily for 5 days) significantly reduced SK channel function in VTA DA neurons 7 days after the last EtOH injection, deemed by the investigators to be post withdrawal (Hopf *et al*, 2007). This loss of SK channel function could lead to increases of *in vivo* VTA DA bursting activity and provide increased incentive salience to EtOH (Hopf *et al*, 2007).

Glutamatergic and GABAergic synaptic modulation of VTA DA firing is also dramatically altered after acute and chronic EtOH exposure and withdrawal. Ethanol is known to inhibit glutamatergic NMDAR function (Lovinger *et al*, 1989; Morikawa and Morrisett, 2010). One injection of 20 mg/kg EtOH caused an enhanced AMPA/NMDA ratio (Figure 5) (Saal *et al*, 2003). This could enhance synaptic strength between VTA DA neurons and their glutamatergic input. However, there are reported strain differences in this response (Wanat *et al*, 2009). Alterations in GABAergic synapses are also mediators of EtOH excitability in VTA DA neurons. One *in vivo* acute injection of EtOH resulted in increased GABAergic transmission, although whether these GABAergic inputs originated from VTA GABAergic interneurons or from GABAergic inputs were not investigated (Melis *et al*, 2002). Bath application of 50 mM EtOH concentrations increased the frequency of GABA-mediated inhibitory post-synaptic currents (IPSCs) via an increased probability of GABA release onto VTA DA neurons from young rats (Theile *et al*, 2008). Furthermore, GABAergic transmission modulates the excitatory effects of EtOH on VTA DA neurons (Theile *et al*, 2010). However, within the VTA itself, *in vivo* activity of GABAergic interneurons have been shown to be inhibited with EtOH administration during recordings via an NMDAR-mediated event (Figure 5) (Stobbs *et al*, 2004). Overall, these differing GABAergic synaptic alterations suggest the possibility of unique populations of GABAergic afferent modulation of VTA DA firing. To date, there are few circuit investigations of the role of glutamatergic and GABAergic synapses from specific afferent inputs from brain regions that are known to regulate VTA DA firing. Given that regions such as the PFC, LDT, LHb, RMTg, and the VP are critical to *in vivo* firing patterns of the VTA DA system, circuit studies into how synaptic plasticity between these regions across the stages of EtOH treatment would provide more insight into how these neurotransmitter systems modulate VTA DA firing after ethanol exposure.

Throughout the years of alcohol research, there have been reports of heterogeneous responses to EtOH. This could be attributed to the reports of VTA DA subpopulations that have differential ion channel/receptor distributions as well as afferent inputs. Rats have shown increased responses to intracranial self-administration (ICSA) of EtOH in the posterior VTA when compared with anterior VTA ICSA of EtOH (Rodd-Henricks *et al*, 2000). This reinforcing behavior seems to be reliant on dopaminergic signaling in the NAc shell and the mPFC, but not the cNAc (Ding *et al*, 2014). Overexpression of HCN channels in the posterior VTA increased ethanol intake (Rivera-Meza *et al*, 2014). Acute exposure to EtOH in the posterior VTA increased VTA DA neuron activity, but decreased VTA DA activity from neurons in the anterior VTA and this was observed in conjunction with a decreased IPSC response in posterior VTA DA neurons and an increased IPSC response in anterior VTA DA neurons (Guan *et al*, 2012). Using TH-green fluorescent protein (GFP) mice, which exclusively express GFP under the TH promoter, investigators found a medial-lateral difference in the response to bath application of EtOH. mVTA DA neurons expressed increased sensitivity to the excitatory effects of bath application of EtOH (as little as 20 mM), while lVTA DA neurons were only excited at higher concentrations (100 mM) (Mrejeru *et al*, 2015). Resolving the diverse functions of VTA DA neuron subpopulations in mediated alcohol reward is critical.

### Nicotine Acts Through Diverse nAChRs on VTA DA Neurons to Regulate Neural Activity

Nicotine, the major psychoactive component of tobacco, acts on endogenous nAChRs throughout the peripheral and central nervous system, including the mesocorticolimbic system (Marti *et al*, 2011; Wonnacott *et al*, 2005). To date, 12 different nAChR subunits (*α*2-*α*10 and *β*2-*β*4) have been identified in the vertebrate brain (Gotti *et al*, 2009; Mansvelder and McGehee, 2002b). These subunits combine in heteromeric formations to create pentameric, ligand-gated ion channels that increases excitability; however, *α*7–*α*9 subunits can make homomeric nAChRs (Boulter *et al*, 1987; Couturier *et al*, 1990a; Couturier *et al*, 1990b; Deneris *et al*, 1989; Wada *et al*, 1988). These varied nAChRs differ in their affinity, conductance, and sensitivity, which overall make up their diverse physiological actions (McGehee and Role, 1995). For example, *α*7-containing nAChRs exhibit higher calcium permeability, yet *β*2-containing nAChRs have higher affinity for nicotine (Laviolette and van der Kooy, 2004; Mansvelder and McGehee, 2002b).

Nicotinic signaling in the VTA is critical to the motivational properties of nicotine. Similar to alcohol, nicotine operates directly on VTA DA neurons to affect neural activity and cause DA release in downstream targets (Calabresi *et al*, 1989; Di Chiara and Imperato, 1988; Grenhoff *et al*, 1986; Nisell *et al*, 1994). Rats will self-administer intravenous (i.v.) nicotine and will decrease their nicotine infusions when given a nAChR antagonist into the VTA, or DA antagonist into the NAc (Corrigall and Coen, 1989a, 1991; Corrigall *et al*, 1994; Corrigall *et al*, 1989b; Exley *et al*, 2011; Maskos *et al*, 2005; Tolu *et al*, 2012). Grenhoff *et al*, 1986 were the first to show that i.p. nicotine (0.5 mg/kg) increases the *in vivo* burst activity of VTA DA neurons of anesthetized rats. In a brain-slice preparation of rat VTA DA neurons that were identified as dopaminergic with classic electrophysiological criteria of inhibition with DA bath application, investigators discovered that 10–100 μM concentrations of nicotine caused increased depolarization, firing rate and inward current in a subset of neurons (77%) recorded, yet 100% of them responded to acetylcholine's ability to increase depolarization, firing rate, and inward current (Calabresi *et al*, 1989). In this study, investigators further discovered that the increase in neural activity occurred in the presence of tetrodotoxin (TTX) and cobalt, confirming that the increase in neural activity can be due to direct actions of nicotine on a subset of VTA DA cells and not just through synaptic modulation (Figure 5).

VTA DA neurons express primarily *α*2–*α*7 and *β*2–*β*4 subunits (Klink *et al*, 2001; Pidoplichko *et al*, 1997). *β*2-containing nAChRs in VTA DA neurons have a high affinity for nicotine, a long desensitization rate and exhibit a strong upregulation during chronic nicotine exposure (Changeux, 2010). VTA DA neurons also express the low affinity *α*7-containing nAChRs, which are more permeable to calcium and may mediate synaptic transmission more than other nAChRs (McGehee and Role, 1995; Seguela *et al*, 1993). Studies performed on transgenic mice have tried to identify the particular nAChRs subunits that encode the reinforcing properties of nicotine (Changeux, 2010; Pons *et al*, 2008; Reperant *et al*, 2010). In particular, VTA *β*2 containing nAChRs have been demonstrated to be key modulators of nicotine's positive reinforcing properties and effects on VTA DA bursting (Faure *et al*, 2014). Mice with a *β*2-KO will not self-administer nicotine (Picciotto *et al*, 1998). Reintroducing the *β*2-subunit into the VTA with specific lentiviruses in *β*2-KO mice restores nicotine's reinforcing properties (Mameli-Engvall *et al*, 2006; Maskos *et al*, 2005; Tolu *et al*, 2012). In addition, genome-wide association studies identified a human polymorphism of nAChRs with *α*3, *α*5, and *β*4 subunits that may underlie a risk for increased nicotine intake and addiction (Wang *et al*, 2009). Knocking out *α*5-subunits in VTA DA neurons caused nicotine's excitatory effects *in vivo* to only occur at higher concentrations of nicotine and mimicking this *α*5-subunit polymorphism in mice led to increased self-administration of nicotine (Morel *et al*, 2013).

An important attribute to heteromeric nAChRs is the rate of desensitization each subunit contributes to, which could contribute to nicotine's strong craving effects. Using a whole-cell electrophysiological assay with nicotine concentrations in a physiological range, Pidoplichko *et al* demonstrated that bath application of 0.1 or 0.5 μM nicotine onto VTA DA neurons in an acute slice preparation, which exhibited TH and *I*_h_, rapidly induced increased depolarization, firing rate and inward current (Henningfield *et al*, 1993; Pidoplichko *et al*, 1997). Interestingly, these investigators discovered that during the bath application of 0.5 μM nicotine, brief acetylcholine pulses, which would normally induce inward currents, were significantly reduced with a downward shift in baseline current suggesting a hyperpolarization of the VTA DA neurons and ultimately a desensitization. After a 1 min bath application, acetylcholine's ability to induce action potentials was also reduced, suggesting a larger desensitization of nAChRs. During a longer bath application (19 min), nicotine caused stronger desensitization to acetylcholine pulses, causing a return to original resting membrane potential, baseline current, and blockade of acetylcholine-induced action potentials. This study showed physiologically relevant mechanisms of nicotinic action on VTA DA neurons, and investigated the role of desensitization in nAChR regulation of dopaminergic activity. This desensitization of dopaminergic activity has been hypothesized to contribute to nicotine craving (Pidoplichko *et al*, 1997).

Researchers have also been focused on how nicotine modulates synaptic transmission onto VTA DA neurons. Although low concentrations of nicotine are enough to stimulate VTA dopaminergic activity intrinsically via nAChRs, particularly through high-affinity nAChRs, this mechanism is not enough to explain how a single injection of nicotine can result in increased DA release in downstream regions lasting for more than an hour (Di Chiara and Imperato, 1988). Interestingly, a single injection of nicotine can cause an increase in the AMPA/NMDA ratio on VTA DA neurons, suggesting a glutamatergic mechanism of nicotine-induced synaptic plasticity (Figure 5) (Saal *et al*, 2003). nAChRs are expressed on both presynaptic glutamatergic terminals in the VTA and post-synaptically on VTA DA neurons, and are key mediators of glutamatergic LTP (Laviolette and van der Kooy, 2004; Mansvelder *et al*, 2002a). Calcium-permeable *α*7-containing nAChRs expressed on presynaptic glutamatergic terminals can induce high calcium influxes to potentiate neurotransmitter release and facilitate synaptic strengthening (glutamatergic LTP) (Figure 5) (Seguela *et al*, 1993). Picciotto *et al*, 1998 hypothesized that the mechanisms underlying the downregulation of self-administration in *β*2-KO mice could be due to a lack of a functional depolarization with nicotine, which may weaken NMDA receptor mediated glutamatergic LTP.

Nicotine also activates GABA neurons, including GABAergic projections to the VTA DA neurons and VTA GABA interneurons, through nAChRs with *α*7 and *α*4*β*2-subunits (Figure 5) (Faure *et al*, 2014). There are a number of mechanisms by which nicotine acts on GABA afferent inputs, VTA GABA interneurons and VTA DA neurons to ultimately increase DA release have been proposed. Here we will briefly discuss two separate hypotheses: the disinhibition of GABA; and the coactivation of VTA DA and GABA neurons in response to nicotine. Bath application of 1 μM of nicotine while recording from *I*_h_-positive VTA DA neurons *in vitro,* using horizontal VTA slices that preserve GABAergic afferents from the NAc and VP, caused increases in IPSC frequency and amplitude, followed by a decrease in frequency in seven out of 11 neurons (Mansvelder *et al*, 2002a). This suggests a desensitization of nicotine on GABA neurons and thus a reduction in inhibitory tone (Mansvelder *et al*, 2002a). The combination of desensitization of GABA tone on a subpopulation of VTA DA neurons, increased glutamate potentiation and a shift to a depolarized state is thought to then shift the major regulatory control of VTA DA neurons to an excitatory state, resulting in prolonged DA release onto downstream targets (Mansvelder *et al*, 2002a). Recently, however, a mechanism identifying coactivation of VTA GABA and DA neurons has been proposed to also increase VTA DA neuron firing and nicotine reinforcement. Anesthetized *in vivo* recordings in mice demonstrated that VTA GABA interneurons do not desensitize to repeated i.v. injections of nicotine (30 μg/kg, an acutely rewarding dose), and that a balance between excitation on both VTA GABA and DA populations shape VTA DA neuron bursting (Tolu *et al*, 2013). These two studies suggest alternate mechanisms of GABAergic regulation of VTA DA neurons based on GABA source (afferents from outside substrate *vs* interneurons in VTA), which highlights the importance of studying specific circuit regulation of subpopulations of VTA DA neurons.

Studies have shown strong heterogeneity in response to nicotine in VTA DA neurons that could be due to intrinsic composition of nAChRs or differential synaptic inputs from a number of cortical and cortical regions (Figure 2) (Ikemoto, 2007; Ikemoto *et al*, 2006). A discovery made by Calabresi *et al*, 1989 demonstrated that less than one third of classically identified VTA DA neurons did not respond to nicotine, which highlights not only the functional diversity of VTA dopaminergic subpopulations, but also the mixed distribution of nAChRs across the VTA. In addition, Pidoplichko *et al*, 1997 discovered that there is large variability in post-nicotine depolarization recovery, acetycholine-induced currents with nicotine bath application.

There seems to be consistent anatomical, possibly projection-specific, disparities in nicotinic responses. Posterior VTA DA neurons display higher activation in response to nicotine than anterior VTA DA neurons (Li *et al*, 2011). In addition, a subset of dorsally located mVTA DA neurons was found to be inhibited by i.v. nicotine (15–90 μg/kg) via D2 receptor activation, while more ventral-laterally located VTA DA neurons were excited (Eddine *et al*, 2015). Methodical investigations into determining whether projection-specific subpopulations of VTA DA neurons differentially express nAChR subunits could further elucidate the role region-specific dopaminergic signaling has in nicotine reward.

### Opioid Modulation of VTA Dopamine Firing via Disinhibition

The endogenous opioid system is composed of internally synthesized opioid peptides that bind to opioid receptors to regulate pain, reward, and stress. Opiates, such as morphine, and opioids, such as heroin, use this endogenous system to mediate their addictive qualities. Of particular interest to the opiate/opioid drug addiction field are MORs. MORs are expressed in a somatodendritic and presynaptic manner and are thought to inhibit neuronal activity either hyperpolarization via GIRKs on the membrane or through attenuation of presynaptic vesicular release (Fields and Margolis, 2015). Morphine and heroin act as agonists to MORs to induce their neurophysiological effects. MORs expressed in the VTA, of which *β*-endorphins have a high affinity for, are crucial to the encoding of a rewarding or hedonic state. Rats will ICSA MOR agonists into the VTA (Bozarth and Wise, 1981a, 1981b). Additionally, systemically administered morphine needs an intact VTA to produce CPP (Olmstead and Franklin, 1997). Systemic administration of morphine or direct infusion of morphine into the VTA both cause DA release in the NAc (Di Chiara and Imperato, 1988; Leone *et al*, 1991).

Although the VTA's role in mediating morphine reward related behaviors is complicated, especially in opiate self-administration (Gratton, 1996), investigators have sought to understand the actions morphine has on VTA DA neuron activity. Increased release of DA in the NAc after morphine is associated with increased *in vivo* spontaneous activity and bursting activity of VTA DA neurons (Gysling and Wang, 1983; Koo *et al*, 2012). Interestingly, investigators identified a population of non-DA cells in the VTA that were suppressed in activity following the morphine administration, which led them to hypothesize that morphine's actions to increase VTA DA activity was indirectly mediated by the suppression of non-DA cells in the VTA (Figure 5) (Gysling and Wang, 1983). In these studies, the investigators identified ‘principal cells' that experienced D2 receptor mediated autoinhibition and that were nonresponsive to [met^5^]-enkephalin. ‘Secondary cells' were thought to be non-DA interneurons because they showed a very narrow action potential waveform and had the opposite response to DA and [met^5^]-enkephalin that principal cells displayed. The hyperpolarization observed by secondary cells was found to be mediated by MORs and an increase in K^+^ conductance. Activation of MORs caused a decrease in the frequency, but not amplitude, of spontaneous IPSCs on VTA DA neurons and, this in turn increased the spontaneous activity of VTA DA neurons. More recently, investigators have further confirmed that this populations of VTA GABA neurons either undergo a somatodendritic hyperpolarization via MOR activation of K^+^ current to reduce VTA GABA firing or they undergo presynaptic terminal inhibition (Johnson and North, 1992a; Steffensen *et al*, 2006). Opioid inhibition of GABA signaling onto VTA DA neurons is not exclusive to VTA GABA interneurons. By virally expressing ChR2 in VP GABA afferents into the VTA, investigators found that light-activated IPSCs on VTA DA neurons were inhibited with MOR agonists. In addition, *in vitro* recordings from retrograde fluorescent beads, that identify VP-VTA GABA neurons, revealed that this GABAergic substrate found to be critical to tonic inhibition of VTA DA neurons *in vivo* is inhibited by MOR agonists (Hjelmstad *et al*, 2013).

The acute synaptic plasticity results of morphine on VTA DA neurons have since been investigated thoroughly, albeit using conventional ways to identify VTA DA neurons that might have tended to be specific for VTA-lsNAc neurons. Acute morphine treatment consistently enhances glutamatergic signaling in VTA DA neurons. A single injection of morphine increases the AMPA/NMDA ratio on VTA DA neurons (Figure 5) (Saal *et al*, 2003). A possible mechanism for this morphine-induced increase in AMPA/NMDA ratio could be because of the insertion of calcium-permeable, GluR2-lacking AMPA receptor subunits (Brown *et al*, 2011). In conjunction with this enhanced excitatory network, morphine also mediates GABAergic synaptic modulation. It was found that within VTA DA neurons that experience D2 receptor-mediated autoinhibition, LTP of GABA synapses (LTP-GABA) is decreased after morphine, a possible mechanism for the increased excitation of VTA DA neurons (Nugent *et al*, 2007). It was recently discovered that weakening GABA synapses through LTD (LTD-GABA) also weakened GABAergic transmission onto VTA DA neurons after a single injection of morphine (Dacher and Nugent, 2011a). Interestingly, morphine may provide a bidirectional control of synaptic plasticity (Dacher and Nugent, 2011b). The effects of chronic morphine have been studied less but are known to also induce plasticity mechanisms that induce reward seeking behaviors, particularly during withdrawal. These mechanisms are have been previously described comprehensively (Mazei-Robison and Nestler, 2012).

Morphine has been also shown to have heterogeneous responses in VTA DA neuron subpopulations that express distinct intrinsic properties and are modulated by differential afferents. These subpopulations of VTA DA neurons have been shown to display heterogeneous and sometimes opposite responses to morphine. When comparing VTA-NAc and VTA-BLA DA neurons, investigators found differential responses to [met ^5^]-enkephalin MOR activation (Ford *et al*, 2006). Recently, subpopulations of VTA DA neurons have been identified to be either directly excited or directly inhibited MOR agonists *in vitro* (Margolis *et al*, 2014). Interestingly, Gysling and Wang, 1983 had observed a differential percent increase over baseline firing in VTA DA neurons after morphine administration. VTA DA neurons that had a slower baseline firing rate had larger percent increases than those cells that had a higher baseline firing rate. We now know that lVTA-lsNAc DA neurons exhibit slower firing frequencies than mVTA-mPFC, mVTA-BLA, and mVTA-cNAc neurons (Chaudhury *et al*, 2012; Lammel *et al*, 2008). If distinct subpopulations of VTA DA neurons do indeed have differential incremental increases in DA firing, this could result in different changes of DA concentrations in downstream targets of the VTA, helping tune behaviors in a finer manner.

The RMTg-VTA-lsNAc circuit is of interest for morphine actions as well. VTA DA neurons that project to the lsNAc are known to receive GABAergic modulation from the RMTg (see above). The GABA neurons in the RMTg express MORs in both their cell bodies and in their presynaptic terminals in the VTA (Sanchez-Catalan *et al*, 2014). These neurons are inhibited by morphine and MOR agonists (Lecca *et al*, 2011; Matsui *et al*, 2014; Matsui and Williams, 2011). The RMTg-VTA-lsNAc circuit could be a critical target for morphine's rewarding actions. Identifying other possible circuit-specific synaptic modulations of VTA DA neurons after acute morphine could be critical to understanding actions on plasticity.

### Cocaine's Actions through Dopamine Transporters and Synaptic Potentiation

Cocaine mediates its effects on the mesolimbic DA system through its reinforcing and rewarding properties. Thus, much work has focused on how cocaine alters this system both acutely and following chronic administration and withdrawal. Cocaine dramatically modulates tonic DA levels in the terminal regions of VTA projections, primarily in the NAc, by binding to DATs and inhibiting its actions (Figure 5). Cocaine attenuates the reuptake of DA into presynaptic terminals and greatly prolongs its action on both D2 auto- and hetero-receptors. This uptake inhibition results in dramatically increased DA concentrations (Ferris *et al*, 2014; Giros *et al*, 1996; Sulzer, 2011). Furthermore, the ability of cocaine to inhibit DATs and elevate DA levels is drastically changed by a history of chronic cocaine history. However, there are contradictory reports of sensitization and tolerance directly at the DAT. Much of this has been attributed to the time course and pattern of cocaine concentrations in the blood between these two models and its effects on DAT function. These differences have been reviewed extensively in a recent publication (Siciliano *et al*, 2015).

In addition to the effects of cocaine on DATs, cocaine also has indirect effects on the presynaptic DA terminal in the NAc. Cocaine-induced elevations in synaptic DA levels also modulate D2-autoreceptor function by promoting their desensitization (Siciliano *et al*, 2015). D2-autoreceptor function is critical to inhibiting DA release and ultimately the regulation of DA tone. Repeated cocaine can result in compensatory alterations in this system that alter basal DA tone, resulting in a hypodopaminergic state where alterations in autoreceptor function and uptake rate produce reductions in DA tone. Interestingly, there is great heterogeneity in the expression and function of DATs and D2 receptors within the subpopulations of VTA DA neurons (Lammel *et al*, 2008). This differential expression of DATs and D2s could lead to the differential release and uptake dynamics in regions of the brain that are known to encode drug-related information for goal directed behaviors.

Because cocaine inhibits uptake, it can potentiate the size and temporal profile of DA transients in VTA projection regions at the presynaptic level. However, in addition to increasing DA transients at the presynaptic level, cocaine also has potent abilities to alter glutamatergic and GABAergic synaptic plasticity on VTA DA neurons. A history of repeated cocaine injections has been shown to increase glutamate in the VTA in response to a cocaine challenge (Kalivas and Duffy, 1998). This increase in glutamate was found to be dependent on functional D1 receptors on glutamatergic afferents in the VTA and may be a key factor to glutamatergic synaptic enhancement on VTA DA neurons (Kalivas and Duffy, 1998). Furthermore, an ultrastructural analysis of glutamatergic terminals in the VTA found increased glutamatergic immunolabeling in cocaine sensitized mice as compared with acutely injected mice (Kozell and Meshul, 2001). In contrast, repeated cocaine injection induced a decrease in D1-mediated GABAergic transmission in the VTA that was regulated by adenosine receptors and cAMP modulation (Bonci and Williams, 1996).

Glutamatergic plasticity on VTA DA neurons is critical to the persistence of drug-seeking behaviors (Engblom *et al*, 2008). A single injection of cocaine was found to increase AMPA/NMDA ratio on conventionally identified VTA DA neurons 24 h after injection (Figure 5) (Ungless *et al*, 2001). Moreover, this single injection of cocaine occluded the induction of high-frequency LTP, suggesting that VTA DA neurons had undergone cocaine-induced LTP (Ungless *et al*, 2001). This effect lasts for 5 but not 10 days after the single injection and was mediated by increased AMPA receptor-mediated currents (Ungless *et al*, 2001). A decrease in NMDA receptor-mediated currents was also discovered after a single injection of cocaine and predicted increased inward rectification and increased AMPA receptor-mediated currents, possibly through the insertion of calcium-permeable AMPA subunits (Mameli *et al*, 2011). The importance of cocaine-induced glutamatergic synaptic plasticity in the VTA is further confirmed by a study that discovered that blocking ionotropic glutamate receptors in the VTA prevents the formation of cocaine CPP (Harris and Aston-Jones, 2003).

Chronic cocaine exerts intrinsic and synaptic plasticity changes across VTA DA neurons. Foundational work discovered that chronic cocaine (10 mg/kg twice daily i.p. injections for 14 days) reduced D2-mediated autoinhibition of VTA DA neurons and increased the number of spontaneously active VTA DA neurons *in vivo* (Henry *et al*, 1989). Investigators discovered that repeated (5–7 days) *in vivo* cocaine administration (15 mg/kg) in rats facilitates NMDA receptor-mediated LTP induction in VTA DA neurons that exhibit a large *I*_h_ (Liu *et al*, 2005). This induction of LTP following repeated cocaine administration was caused by a reduction of GABA_A_ receptor activity, as determined by the reduction of the amplitude of GABA-mediated currents (Liu *et al*, 2005). These findings suggest that chronic cocaine results in decreased GABAergic inhibition of VTA DA neurons. Withdrawal (10–15 days) from the same regimen of repeated cocaine administration allowed for the induction of NMDA receptor mediated LTP after weak presynaptic stimulations, which was not observed in saline-treated rats or cocaine-treated rats that experienced a 24 h withdrawal. This suggests a sensitization of VTA DA neurons following withdrawal (Pu *et al*, 2006). Interestingly, brain-derived neurotrophic factor (BDNF) was found to be a critical mediator of this sensitivity. Bath application of BDNF onto VTA slices before, but not during, weak presynaptic stimulations facilitated LTP of VTA DA neurons in saline-treated rats and rats that had experienced only 24 h of withdrawal, an effect that was further determined to be dependent on BDNF's effects post-synaptically and was occluded in rats that had 10–15 days of withdrawal post-synaptically as well (Pu *et al*, 2006).

Current research has identified heterogeneous responses to cocaine to parse out the subpopulations of VTA DA neurons mediating the rewarding properties of cocaine. It was demonstrated that i.v. injection of cocaine in anesthetized rats induced a partial inhibition of activity in VTA DA neurons that were identified to project to the NAc (Einhorn *et al*, 1988). However, it was also observed that *in vivo* injections of cocaine in awake rats differentially altered the activity of VTA DA neurons, with only 14% exhibiting a decrease of firing rate and bursting activity and a significant percent of the population experiencing an increase in activity (Koulchitsky *et al*, 2012). Recently, in a thorough anatomical and immunohistochemical investigation, investigators identified that a majority of VTA DA neurons that were inhibited by cocaine resided in the posterior VTA, while those that were excited were more anterior in the VTA during anesthetized *in vivo* recordings (Mejias-Aponte *et al*, 2015).

In addition, it was discovered that one injection of cocaine-induced increases in AMPA/NMDA ratios 24 h later, in VTA DA neurons that specifically project to the lsNAc and msNAc (Lammel *et al*, 2011). Interestingly, the changes observed in those VTA DA neurons that project to the msNAc were persistent up to 21 days after single cocaine injection, which correlates with findings of larger increases in DA concentrations in the msNAc (Aragona *et al*, 2008). Surprisingly, no changes in synaptic strength were observed in VTA DA neurons that project to the mPFC; however, changes were observed in this projection after an aversive formalin injection into the paw (Lammel *et al*, 2011). Based on circuit tracing studies, we now know specific afferent inputs could underlie these differential stimulus responses. *In vivo* modulation of differential glutamatergic afferent inputs onto VTA DA neurons using optogenetically induced LTP/LTD or using designer receptors exclusively activated by designer drugs to alter dopaminergic activity following cocaine injection would provide more insight into how circuit-specific actions lead to cocaine-induced drug plasticity.

## HYPOCRETINS ACT AS NEUROMODULATORS OF DRUG-INDUCED PLASTICITY ON VTA DOPAMINE NEURONS

Hcrts has an important role in mediating the associative and cue-induced reinstatement properties of multiple classes of drugs, including alcohol, morphine, and cocaine. Transgenic mice that have a KO of the *prepro-hcrt* gene do not establish a CPP to cocaine (Narita *et al*, 2006). Systemic injections of the hcrtR1 antagonist SB-334867 (SB) before the test session of morphine CPP show reduced preference for the morphine conditioned side, demonstrating hcrt involvement in behaviors involving drug associations (Harris *et al*, 2005). Hcrt is also important for cue-induced drug reinstatement following extinction. Systemic blockade of hcrtR1-reduced ethanol seeking and cocaine seeking (Lawrence *et al*, 2006; Smith *et al*, 2009). The VTA is a critical neural substrate for modulating these actions. Infusion of SB into the VTA directly reduced morphine CPP (Narita *et al*, 2006). In addition, LH-VTA neurons were demonstrated to have robust correlations of c-fos induction with morphine CPP (Aston-Jones *et al*, 2009a; Aston-Jones *et al*, 2009b). These investigations found no differences in LH projections that preferentially synapse in the rostral or caudal VTA.

Hcrts are important mediators of synaptic plasticity on VTA DA neurons. Borgland *et al* have consistently demonstrated a crucial role for hcrtR1 in mediating drug-induced synaptic actions. To note, these studies have used the conventional criteria of DA neuron identification for their studies. Using a hcrtR-1 antagonist before cocaine injections for 5 days was shown to block cocaine's potentiation of the AMPA/NMDA ratio and is also critical to the induction of the locomotor sensitization effects of cocaine (Borgland *et al*, 2006). Recently, it was discovered that hcrtR1 is also critical for the synaptic actions observed following morphine administration. Blockade of hcrtR1 on VTA DA neurons prevents AMPA/NMDA potentiation, prevents morphine-induced increases in AMPA receptor-mediated current frequency and amplitude, and also prevents morphine-induced increases in presynaptic glutamate release (Baimel and Borgland, 2015). Interestingly, in the same study, they also discovered that hcrtR1 was important for the morphine-induced decreases in GABA release onto VTA DA neurons (Baimel and Borgland, 2015).

Modulation of hcrt signaling seems critical for the drug-induced increases of DA in downstream targets. Morphine's ability to increase DA release in the NAc is reduced in prepro-hcrt KO mice (Narita *et al*, 2006). Infusion of hcrt1 directly into the VTA potentiated the cocaine-induced DA release in the NAc (Espana *et al*, 2010). Concurrently, blockade of hcrtR1 decreased this release (Espana *et al*, 2010). Investigations into the projection-specific expression of hcrt-Rs would be critical in understanding how different subpopulations of VTA DA neurons are modulated by their differential synaptic inputs, particularly on VTA DA neurons that project to the mPFC or the BLA.

## STRESS AND DRUG INTERACTIONS

Stressful events are known to enhance the reinforcing properties of drugs and influence drug-seeking behaviors in both humans and non-human animal models (Erb *et al*, 1996; Kalivas and McFarland, 2003; Piazza and Le Moal, 1996; Sanchis-Segura and Spanagel, 2006). Interestingly, withdrawal from drugs of abuse is known to activate neural circuits associated with stress response and has been thoroughly reviewed elsewhere (Koob, 2008; Koob *et al*, 2014). However, here we will focus on how the experience of a stressful or aversive can cause alterations for the mesocorticolimbic system to influence drug-associated behaviors and drug-induced plasticity.

Mild stressors, such as footshock or food restriction, have been shown to cause larger reinforcement of heroin, increased cocaine sensitization and higher cocaine CPP (Goeders and Guerin, 1994; McLaughlin *et al*, 2003; Rouge-Pont *et al*, 1995; Shaham and Stewart, 1994). Furthermore, psychological stressors, such as social isolation, restraint stress, or social defeat stress, can increase administration of drugs of abuse (Croft *et al*, 2005; Schenk *et al*, 1985; Shaham, 1993). The VTA has been identified as an important mediator of these behaviors through a number of means that include interaction of VTA DA neurons with CRF, GCCs, and differential synaptic inputs that are responsive to stress. Importantly, the VTA is critical for stress-induced reinstatement of drug-seeking behavior (Graziane *et al*, 2013; McFarland *et al*, 2004). Further understanding how both stressful stimuli and drug-induced plasticity mechanisms converge on VTA DA neurons is critical. Indeed, recently, investigators discovered that VTA DA neurons that were excited by cocaine administration were also more likely to be responsive to a typically aversive footshock stimulus (Mejias-Aponte *et al*, 2015).

One way that stress can affect drug-induced plasticity is through the stress-related neuropeptide, CRF. CRF influences VTA DA activity and is a regulator of plasticity (see above). Blockade of CRF-R1 in the VTA prevents cocaine-induced DA increases in the NAc (Lodge and Grace, 2005). Mild footshock induces reinstatement of DA activation with glutamate release and CRF release in the VTA in cocaine-experienced animals (Wang *et al*, 2005). CRF-Rs and CRF-BP have been shown to be critical in the VTA since non-specific antagonism of both receptors prevents footshock events, and CRF's effects to potentiate glutamatergic NMDA receptor currents is dependent on CRF-R2 and CRF-BP function (Ungless *et al*, 2003; Wang *et al*, 2007). However, there have been reports suggesting that cocaine experience is necessary to induce long-lasting changes in plasticity for stress or CRF to have its reinstatement actions (Wise and Morales, 2009). Yet, mice that binge drink alcohol undergo CRF-R1 mediated NMDA receptor current enhancement, suggesting an acute action from binging (Sparta *et al*, 2013). The differential expression of the two CRF receptors in the VTA also calls into the question: which subpopulations of VTA DA neurons are mediating these aversive events? CRF was shown to have D2 autoreceptor desensitization actions in the subpopulation of VTA DA neurons that express D2, leaving a subpopulation to be investigated for other possible CRF intrinsic actions (Nimitvilai *et al*, 2014).

Stressful events can trigger the synthesis and release of GCCs, which have actions on the mesocorticolimbic system (see above). GCCs released during stress have also been shown to be influential in the reinforcing properties of drugs of abuse as well as in relapse behaviors. Blocking corticosterone synthesis during stressful food restrictions was found to block sensitization to cocaine and morphine, suggesting a GCC role in inducing susceptibility to drug reward (Deroche *et al*, 1992; Rouge-Pont *et al*, 1995). Indeed, there have been many investigations exploring the mechanisms by which GCCs mediate individual vulnerabilities to drug reinforcement, which have been reviewed (Piazza and Le Moal, 1996). Morphine's actions in the VTA to induce locomotor responses were also demonstrated to be dependent on GR signaling (Marinelli *et al*, 1998). GCC modulation of afferent plasticity in coherence with drug-induced synaptic plasticity could be a critical mediator of enhanced behavioral responses to drugs of abuse.

The VTA, with its inputs from cortical and subcortical structures, acts as a hub for a number of neural afferents that converge to encode information about intrinsic and external states. As stated above, many researchers are discovering input-specific control of subpopulations of VTA DA neurons. Studies that investigate how differential afferents regulate VTA DA response in the face of stress experience and drug use are now crucial. Since Lammel *et al*'s work suggest that mPFC and lsNAc, but not msNAc, projecting VTA DA neurons act as substrates for encoding different stimuli, it would be interesting to see how a convergence of typically rewarding (drugs of abuse) and aversive stimuli alter plasticity mechanisms. One source of afferent control is the vSub, which is thought to be an important neural substrate in the activation of VTA DA neurons in contextually relevant situations due to its influence on VTA DA neuron population activity (Belujon and Grace, 2015). Acute restraint stress causes sensitization to psychomotor effects of the psychostimulant, amphetamine, concurrent with increases in VTA DA neuron population activity, which is known to be disinhibited by the vSub-NAc-VP-VTA circuit (Valenti *et al*, 2011). This increase in VTA DA activity is dependent on the vSub input (Belujon and Grace, 2011; Valenti *et al*, 2011). Recently, a new vSub circuit linking the vSub-BNST-VTA circuit was identified to be critical in cocaine's locomotor affects and in the induction of increased activity in a subpopulation of VTA DA neurons (Glangetas *et al*, 2015). Further investigation into how relay circuits of fast-acting neurotransmitters work in conjunction with stress- and drug-responsive neuromodulators would also elucidate neural circuit mechanisms of stress context-associated drug-induced plasticity in the VTA.

## DISCUSSION

We have reviewed the distinct subpopulations of projecting VTA DA neurons, the differential drug-induced plasticity mechanisms on VTA DA neurons and highlighted the necessity for a better understanding of these mechanisms across all subpopulations of VTA DA neurons. The mesocorticolimbic system is a critical mediator of drug abuse. However, the VTA sends functionally distinct DA projections to its targets. Recent advances in circuit dissecting techniques, projection- and cell-specific molecular profiling and *in vivo* imaging of cell projection-specific neuronal activity have opened up new avenues into addressing these issues. In addition, the call for better transgenic mouse lines that allow for dopaminergic specific expression of GFP or Cre-recombinase will help the field further.

## FUNDING AND DISCLOSURE

The authors declare no conflict of interest.

## Figures and Tables

**Table 1:**
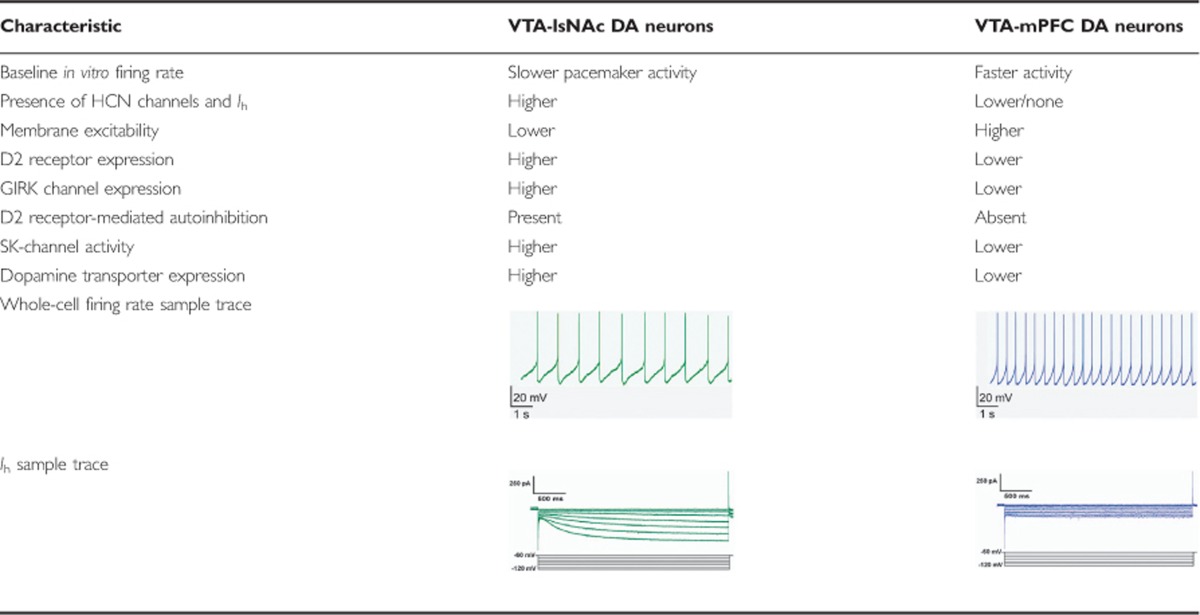
Distinct Electrophysiological Properties of VTA-IsNAc and VTA-mPFC DA Neurons

**Table 2:** VTA Output and Input Circuits and the Related Studies

**Projection**	**Reference**
*VTA output*	
DOPAMINEGIC: VTA-lateral shell NAc, VTA-medial shell NAc, VTA-NAc core, VTA-BLA, and VTA-mPFC	Lammel *et al*, 2008; Chaudhury *et al*, 2012; Friedman, *et al*, 2014
GABAergic: VTA-NAc	Omelchenko and Sesack, 2005
	
*VTA input*	
NAc-VP and VP-VTA	Kalivas *et al*, 1993; Wu *et al*, 1996; Floresco *et al* 2003; Zahm and Heimer, 1990
LHb-RMTg-VTA	Matsumoto and Hikosaka, 2007; Lammel *et al*, 2012
LHb-VTA-PFC	Lammel *et al*, 2012
BNST-VTA	Georges and Aston-Jones, 2001; Georges and Aston-Jones, 2002; Geisler and Zahm, 2005; Jennings *et al*, 2013
LH-VTA	Peyron *et al*, 1998; Fadel and Deutch, 2002
RMTg-VTA	Barrot, 2014; Sanchez-Catalan *et al*, 2014
LC-VTA	Mejias-Aponte *et al*, 2009
LDT-VTA	Forster and Blaha, 2000; Lodge and Grace, 2006; Omelchenko and Sesack, 2005; Lammel *et al*, 2011, 2012
PPTg-VTA	Clements and Grant, 1990; Oakman *et al*, 1995; Floresco *et al*, 2003
VTA GABA projecting neuron-NAc	Omelchenko and Sesack, 2005
CeA-VTA	Gonzales and Chesselet, 1990; Wallace *et al*, 1992
mPFC-VTA, OFC-VTA	Gariano and Groves, 1988; Sesack and Bunney, 1989; Carr and Sesack, 2000b; Takahashi *et al*, 2011
